# Novel Fabrication Process for Integration of Microwave Sensors in Microfluidic Channels

**DOI:** 10.3390/mi11030320

**Published:** 2020-03-19

**Authors:** Juncheng Bao, Tomislav Markovic, Luigi Brancato, Dries Kil, Ilja Ocket, Robert Puers, Bart Nauwelaers

**Affiliations:** 1 KU Leuven, Div. ESAT-TELEMIC, Kasteelpark Arenberg 10, 3001 Leuven, Belgium; Tomislav.Markovic@imec.be (T.M.); ilja.ocket@imec.be (I.O.); bart.nauwelaers@kuleuven.be (B.N.); 2 IMEC, Kapeldeef 75, 3001 Leuven, Belgium; 3 KU Leuven, Div. ESAT-MICAS, Kasteelpark Arenberg 10, 3001 Leuven, BelgiumDries.Kil@kuleuven.be (D.K.); robert.puers@kuleuven.be (R.P.)

**Keywords:** micro-fabrication, microelectromechanical systems (MEMS), microfluidics, microwave dielectric sensing

## Abstract

This paper presents a novel fabrication process that allows integration of polydimethylsiloxane (PDMS)-based microfluidic channels and metal electrodes on a wafer with a micrometer-range alignment accuracy. This high level of alignment accuracy enables integration of microwave and microfluidic technologies, and furthermore accurate microwave dielectric characterization of biological liquids and chemical compounds on a nanoliter scale. The microfluidic interface between the pump feed lines and the fluidic channels was obtained using magnets fluidic connection. The tube-channel interference and the fluidic channel-wafer adhesion was evaluated, and up to a pressure of 700 mBar no leakage was observed. The developed manufacturing process was tested on a design of a microwave-microfluidic capacitive sensor. An interdigital capacitor (IDC) and a microfluidic channel were manufactured with an alignment accuracy of 2.5 μm. The manufactured IDC sensor was used to demonstrate microwave dielectric sensing on deionized water and saline solutions with concentrations of 0.1, 0.5, 1, and 2.5 M.

## 1. Introduction

Microfluidic technology has been developed to manipulate very small amounts of liquid in micrometer-scale channels [1]. In the past decade, researchers have developed a powerful fluidic manipulation tool box including devices such as pumps [2], mixers [3], sorters [4], and valves [5]. Using those powerful tools, biomedical assays can be implemented in a much smaller scale compared to traditional methods. This enables a number of applications such as single cell analysis [6], high throughput molecular synthesis and chemical production [7], cancer cell detection [8], and targeted sequencing [9].

Microwave technology is a powerful tool for biomedical application that enables label-free sensing and broadband spectroscopy for cells, tissues, and proteins [10]. In recent years, a new subdomain called microwave-micofluidics has gained popularity, which integrates microwave circuits and microfluidic channels to provide a new superior tool for biomedical applications, including nanoliter bioliquid broadband spectroscopy [10,11], single cell broadband spectroscopy [12,13], liquid mixture sensing [14,15], flow cytometry [16], and microwave heating for continuous [17,18] and digital [19,20] microfluidic applications, such as microchip-based polymerase chain reaction (PCR) [21].

Polydimethylsiloxane (PDMS)-based microfluidic devices have broad applications in biological studies, because of its low cost, non-toxicity to cells, permeability to gases [22], and chemical compatibility with various of solvents solvents [23]. Soft lithography [24] technology is commonly used to fabricate PDMS microfluidic channels. However, the alignment of the fabricated microfluidic channels with the metal layer on wafer to realize microwave devices can be critical when integrating them. The misalignment between these two layers leads to the dimensional difference between the fabricated device and the designed device. Moreover, this will result in different behaviors between the fabricated devices and the designed devices, which is more significant when the working frequency of the device increases. Since physical dimensions of the misalignment increase relative to wavelength as frequency increase. Furthermore, the alignment accuracy sets a limitation of the resolution of the fabricated microwave-microfluidic device [25], which makes it critical for the down scaling of the devices for applications such as single cell measurements. Research has been done to improve the alignment accuracy either between PDMS fluidic layer and silicon or glass devices or between multi-layer PDMS fluidics structures. A customized desktop aligner has been reported, achieving multilayer PDMS microfluidic structures with an accuracy of 20 μm cm^−1^ [25]. The shrinkage of PDMS needs to be taken into account to increase the alignment accuracy [26]. In addition, the shrinkage of PDMS is related to the curing condition of the PDMS, which makes it more difficult to control. A sandwich mold fabrication process was used to achieve an alignment accuracy of 15 μm [27]. Achieving a better alignment is a necessary requirement to further down scale the microwave-microfluidic applications. We hereby present a novel technique for fabricating microfluidic channels with a lithographically defined well-aligned dissolvable photoresist mold. This method enables the integration of microwave circuits and microfluidic channels with micrometer range alignment without using customized aligner [25]. Molding PDMS directly on the substrate also eliminates the need for an extra bonding step of the PDMS channels. An interdigital capacitor (IDC)-based microwave sensor in microfluidic channel was designed and fabricated with the novelly proposed fabrication process. It was used to distinguish saline solutions with different concentrations.

## 2. Fabrication Prosess

**Figure 1:**
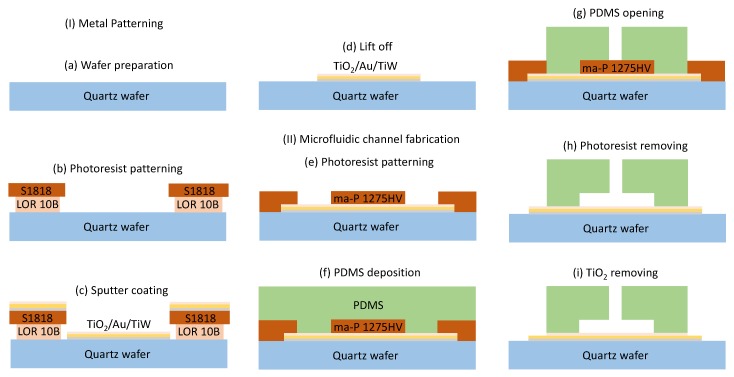
The proposed process to integrate microwave device and microfluidic channel. (**a**) Prepare the wafer for the process. (**b**) Pattern the double layer photoresis. (**c**) Sputter coat TiW, Au, and TiO_2_, successively. (**d**) Lift off in NMP. (**e**) Pattern sacrificial photoresist layer using the EVG620 automated mask aligner. (**f**) Deposit and cure polydimethylsiloxane (PDMS) on the whole wafer. (**g**) Punch liquid access hole with a biomedical perforator and remove unwanted PDMS with a micro scalpel. (**h**) Soak the wafer in acetone to remove the sacrificial photoresist layer. (**i**) Remove TiO_2_ not covered by PDMS layer using RIE.

The above-mentioned devices are fabricated using a two-step process. First, the microwave circuit is patterned onto the substrate. Afterwards, microfluidic PDMS channels are fabricated on top of the microwave circuit (Figure 1).

Fused silica wafers (JS2, Microchemicals GmbH, micro roughness less than 1 nm) were chosen as substrates due to their low microwave loss factor and cost. The transparency of the material allows simultaneous monitoring of the fluidic channel from both the top and the bottom of the sample. The following workflow was applied.

Fabrication of the microwave circuit:

- Four-inch fused silica wafers are thoroughly cleaned using piranha etchant (4 H_2_SO_4_:1 H_2_O_2_) to remove all organic contaminants. Afterwards, a HF-dip (2% HF) is performed, followed by a rinse in DI water. The substrate is dried using purified nitrogen.
- Reactive Ion Etching (RIE) (JLS design Ltd) is used to increase the surface roughness of the wafers, increasing adhesion with the photoresist used for patterning. The RIE treatment lasts for 2 min with a chamber pressure of 100 mT, O_2_ flow of 40 sccm, and RF power of 50 W. This treatment is proved to be crucial in achieving a high fabrication yield for sub 10-μm structures, since it reduces the failure caused by delamination of these photoresist structures during photoresist development.
- A metal stack consisting of 50-nm-thick TiW adhesion promoting layer and 400-nm-thick Au is deposited by sputter coating (Blazers) on a lithographically patterned photoresist bilayer (LOR10B/S1818) [28]. To ensure good adhesion with the PDMS channels fabricated in the following steps, a layer of TiO_2_ having a thickness in nanometer range is sputtered onto the gold. The lift-off is completed by soaking the wafers in n-methyl-2-pyrrolidone (NMP) overnight at room temperature.

Fabrication of the PDMS microfluidic channels:

- A 20-μm thick layer of sacrificial ma-P 1275HV [29] photoresist is spin coated onto the substrate (containing the microwave circuit) for 1 min with a speed of 1000 rpm. The wafer is soft baked for 5 min at 120 °C, after the photoresist is allowed to relax for 20 min to provide sufficient water molecules, which are necessary for the desired photoreaction during the exposure.
- The photoresist is patterned through a chromium mask using the EVG620 automated mask aligner to achieve an alignment accuracy up to 2 μm.
- After development, a RIE treatment is performed to improve the adhesion of the substrate with the PDMS. The RIE treatment lasts for 1 min with a O_2_ flow of 40 sccm, RF power of 100 W, and chamber pressure of 20 mT.
- The base and curing agents of the PDMS (Sylgard 184) are mixed in a 10:1 ratio. The mixture is degassed applying a pulsating vacuum for 15 min, and 7 g of the degassed PDMS is poured on the wafer resulting in a 1-mm-thick layer. Curing is done at a temperature of 80 °C for 1 h to avoid reflowing of the photoresist.
- After complete curing of the PDMS, a biopsy punch [30] (1.5-mm diameter) is used to punch holes to enable connection to the microfluidic channels. The PDMS covering the photoresist protecting the microwave circuit is cut carefully under microscope with micro scalpel.
- The wafer is soaked in acetone for 3 h to remove the sacrificial photoresist.
- Finally, the TiO_2_ not covered by the PDMS layer is removed by a 2 min RIE with a 10 sccm Ar flow [31] to provide electrical connection for microwave signal. The chamber pressure is set to 30 mT and the RF power is 150 W.

The core idea of the above method is to build the lithographic defined and aligned sacrificial photoresist mold to fabricate microfluidic channels in PDMS. The alignment accuracy of this process is only limited by the accuracy of the mask aligner. It does not require custom-made aligners and the alignment is not influenced by the shrinkage ratio of the PDMS since no peel-off from the mold is needed. The used sacrificial photoresist is chosen based on the dimensions of the fluidic channels. Ma-P1275HV is a positive photoresist that can be deposited in 10–50-μm-thick layers in a single coating by tuning the spin coating speed [29]. A side wall up to 87° can be achieved. These make it a proper photoresist for our application. In this process, directly curing the PDMS on the substrate ensures strong adhesion between the two layers. During the sacrificial photoresist removing step, acetone can flow between the gold and PDMS if the gold layer is not covered with TiO_2_. This can cause the failure of the entire process. The time needed to dissolve the sacrificial photoresist mold depends on the length and height of the fabricated fluidic channel and the total thickness of the PDMS layer. Typically, the fabrication process of the fluidic channels can be completed in one day. This process constitutes a valid alternative to soft lithography, eliminating the need for plasma boding of PDMS to the substrate.

## 3. Interdigital Capacitor Sensor Design

**Figure 2:**
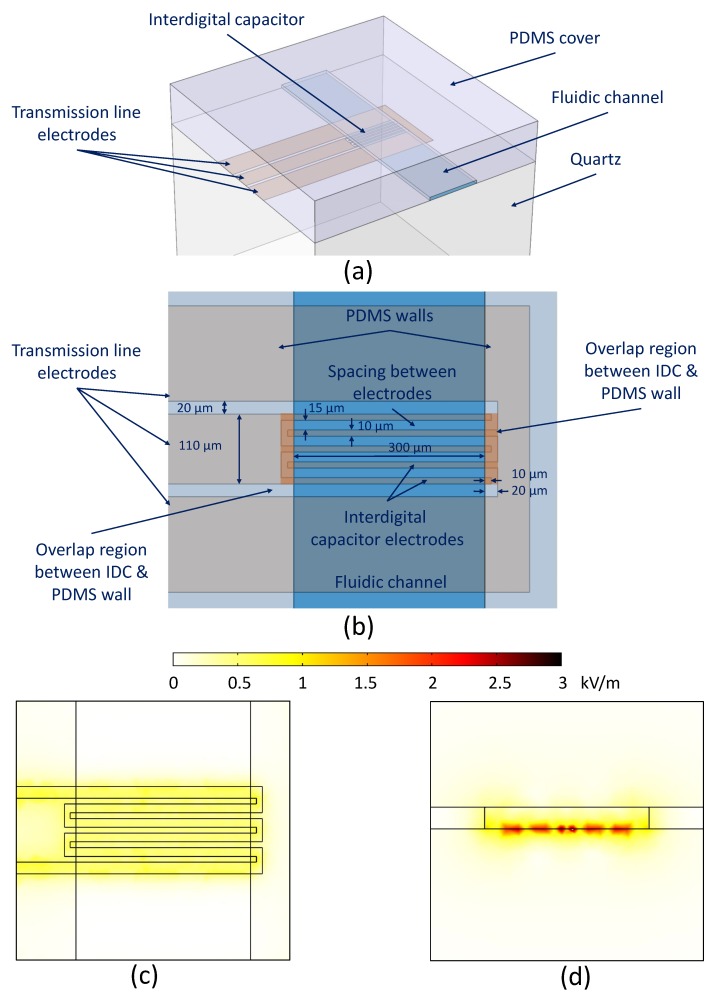
(**a**) A 3D model of the proposed IDC sensor. (**b**) Top view of the proposed IDC sensor with designed diameters. (**c**) Top view of the electrical field distribution at 1 GHz 10 μm above the surface of quartz. (**d**) Side view of the electrical field distribution at 1 GHz across the IDC fingers in the middle of the liquid channel.

The proposed fabrication process was used to fabricate a microwave-microfluidic device for dielectric sensing of liquid in a microfluidic channel. The sensing device was chosen to be an interdigital capacitor (IDC) loaded by the liquid flowing through the channel placed over the IDC, as illustrated in Figure 2a. The sensing device based on the IDC topology was chosen to evaluate the fabrication process as IDCs have been previously applied in many investigations [17,20,32], which have proved a great potential of IDCs in microwave-microfluidic investigations.

The capacitance of the IDC depends on the physical dimensions and mutual spacing of the IDC electrodes and the electric permittivity of substrate and the material loaded on top of the IDC electrodes [33]. By changing the electric permittivity of the material loaded on top of IDC electrodes, the capacitance of IDC is changed accordingly. This change can be measured with VNA; thus, the permittivity change of the loaded material can be sensed.

The design space of the IDC consists of the width of signal and ground fingers and their mutual spacing, as indicated in Figure 2b. These dimensions are often sized to obtain desired electric field distribution and a capacitance value of the IDC. In this design case, the design goal was to keep these dimensions as small as possible to benchmark the proposed manufacturing process. Therefore, IDC design dimensions were set to 10 μm for the width of IDC electrodes and 15 μm for their mutual spacing. The length of the IDC covered by liquid was set to 300 μm. Additionally, to test the positioning of the fluidic channel to the IDC, an overlap of 20 μm between the IDC electrodes and PDMS channel walls was chosen, as illustrated in Figure 2b. The accurate positioning allows the correlation between the simulation model of the IDC sensor and manufactured devices, which consequently helps in accurate de-embedding of feeding transmission lines, precise extraction of the IDC capacitance value, and modeling of the IDC sensor based on electromagnetic simulations and conformal mapping [33]. The designed IDC layout is presented in Figure 2b.

**Figure 3:**
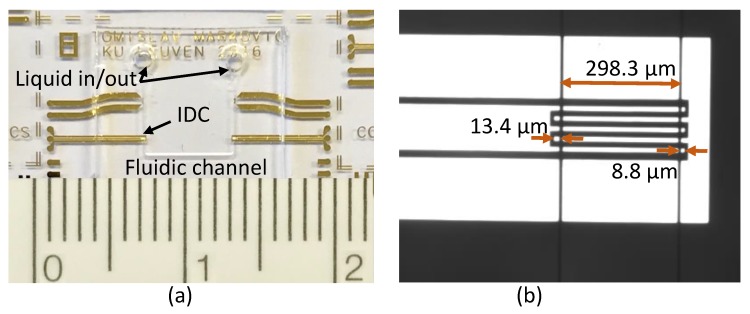
(**a**) Image of the fabricated sensor. (**b**) Microscope image of the fabricated IDC with measured dimensions.

The designed IDC provides a capacitance value of 0.3 pF when DI water is loaded at 1 GHz. The 2D spatial distribution of the electric field in the middle of the channel at 1 GHz was calculated using COMSOL Multiphysics, as presented in Figure 2c,d, and shows limited gradients along the IDC sensor width and length. An image of the manufactured device and a microscope image of the IDC sensor are shown in Figure 3a,b, respectively. Sensofar S lynx compact 3D surface profiler was used to measure the distance between the end of the capacitor finger and the edge of the fluidic channel, as well as the length of the fluidic channel, to quantify the alignment accuracy of the fabricated device. As shown in Figure 3b, the distance between the left finger end and channel edge is 13.4 μm, and the distance of the right side is 8.8 μm. The measured channel width is 298.3 μm, which is 1.7 μm shorter than the designed value of 300 μm. The fluidic channel is around 2.5 μm shifted to the right side, which is less than the margin of 10 μm between the end of the capacitor finger and the channel edge set by the sensor design. In conclusion, the proposed fabrication process accuracy is sufficient to realize the IDC design and avoid the liquid flow over the non-uniform fringing field region, which is the overlap region of 20 μm in our case.

## 4. Measurements

**Figure 4:**
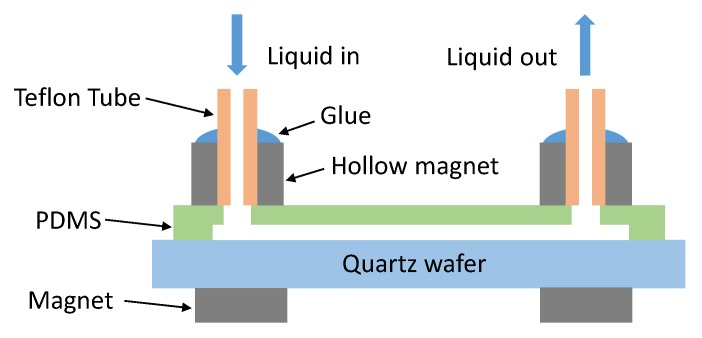
Magnets liquid interconnection between fluidic pump and microwave microfluidic chip.

**Figure 5:**
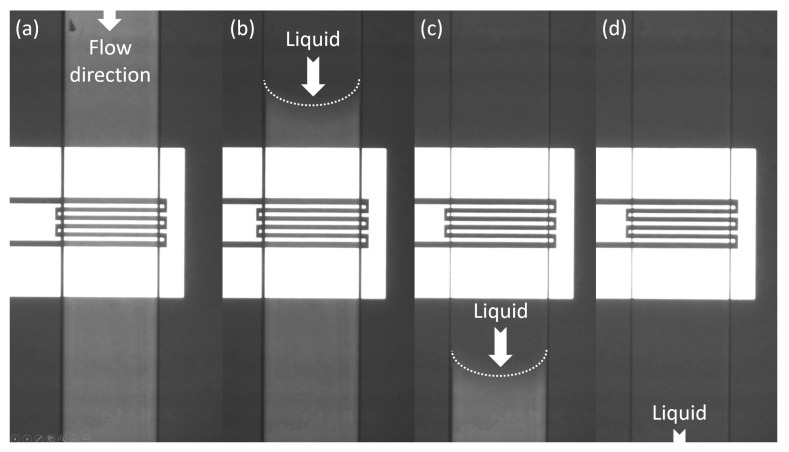
Microscope photograph of the fluidic channel filled by DI water with a flow from top to bottom: (**a**) eEmpty channel; (**b**) partially filled channel; (**c**) partially filled channel; and (**d**) filled channel.

Magnets were used to build up the interconnection between the fluidic pump and the proposed microwave microfluidic chip [34], as shown in Figure 4. A Teflon tube (Elveflow) having a 1/16-inch outer diameter and 1/32-inch inner diameter was inserted into a hollow magnet (Amazing Magnets). The hollow magnet has an outer diameter, inner diameter, and thickness of 1/4 inch, 1/16 inch, and 1/4 inch, respectively. Loctite super glue was used to fix and seal the Teflon tube to the hollow magnet. Then, the tube glued magnet was aligned with the liquid access hole on the chip and held in place by another magnet (Amazing Magnets) at the opposing side of the substrate. The magnet used to hold the tube inserted hollow magnet has diameter and thickness of 1/4 inch an 1/32 inch, respectively. Since PDMS is soft, no sealing gasket needed to be applied to the surface of the tube glued hollow magnet contacting the fluidic chip, as shown in [34]. The holding force of the magnet liquid connectors can be tuned by the number of magnets on the opposing side of the substrate. These realize an easily replaceable fluidic interconnection and no thick PDMS layer is needed to fix the Teflon tube. The Teflon tube is connected to a Fluigent MFCS-EZ microfluidic flow control system to flush different liquids in the microfluidic channels with a well controlled pressure. Figure 5 shows the channel filling by deionized (DI) water flowing from top to bottom without any leakage. The channel having a total length of 15 mm was filled completely in around 1 s when a pressure of 100 mBar was applied. The maximum applicable pressure for the magnetic fluidic connection depends on the applied magnetic force and total thickness of the chip and PDMS. As investigated in [34], a magnetic liquid connector having a PDMS sealing gasket can stand a pressure up to 1700 mBar. During our experiments, no leakage was observed with both the magnetic liquid connector and the fluidic channel when the pressure of the pump was set to 700 mBar.

**Figure 6:**
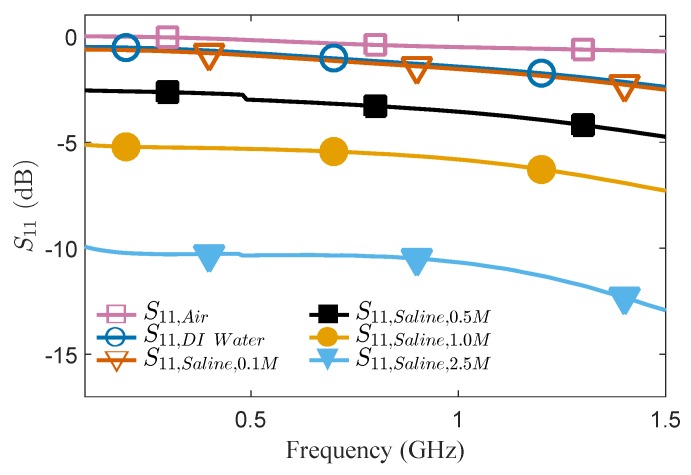
Measured S_11_ of the proposed IDC sensor loaded with saline solutions of different concentrations.

With the proposed IDC sensor, microwave-microfluidic sensing was demonstrated using DI water and saline solutions in concentrations of 0.1, 0.5, 1, and 2.5 M. Liquid solutions were flushed into the fluidic channel and microwave response of the liquid loaded IDC was measured using a vector network analyzer (Agilent E8361A).The separation between the edge of the magnetic fluidic connector and the IDC was designed to be 2.5 mm to allow the seamless IDC operation and optical investigation. The measured signals, namely reflection coefficients of the IDC, represent the ratio of reflected and incident waves determined by dielectric and conductive parameters of the liquid located over the IDC. As shown by the data in Figure 6, the non-resonant IDC sensor allows the differentiation of different saline concentrations starting from 0.1 M in a wide frequency range.

## 5. Conclusions

In this paper, a novel fabrication process integrating microwave circuits and microfluidic channels in PDMS is proposed. A well-aligned sacrificial photoresis layer is used to serve as the mold to fabricate PDMS microfluidic channels. With this fabrication process, an alignment accuracy of 2.5 μm between the metal layer and PDMS microfluidic layer is achieved, by using EVG620 automated mask aligner. An IDC sensor embedded in a PDMS microfluidic channel was designed and fabricated to validate the alignment accuracy of the proposed fabrication process. This process enables us to further down scale microwave microfluidic applications to achieve single cell sensing, microwave sensing, and heating for sub-nanoliter volume.

## Abbreviations

The following abbreviations are used in this manuscript:

DI	deionized
IDC	Interdigital capacitor
NMP	N-methyl-2-pyrrolidone
PCR	polymerase chain reaction
PDMS	Polydimethylsiloxane
RIE	Reactive Ion Etching
